# Supplementary material for: A multi-technique approach for improving summary polar diagrams
Source: iScience. 2026 Aug 24;29(9):117044. doi: 10.1016/j.isci.2026.117044 (PMC13542598; doi:10.1016/j.isci.2026.117044)
Supplement: Supplementary file 1 [file mmc1.pdf]

iScience, Volume *29*

Supplemental information

A multi-technique approach for improving summary polar diagrams

Aleksandar Anžel, Zewen Yang, and Georges Hattab

SUPPLEMENTAL FIGURES AND TABLES

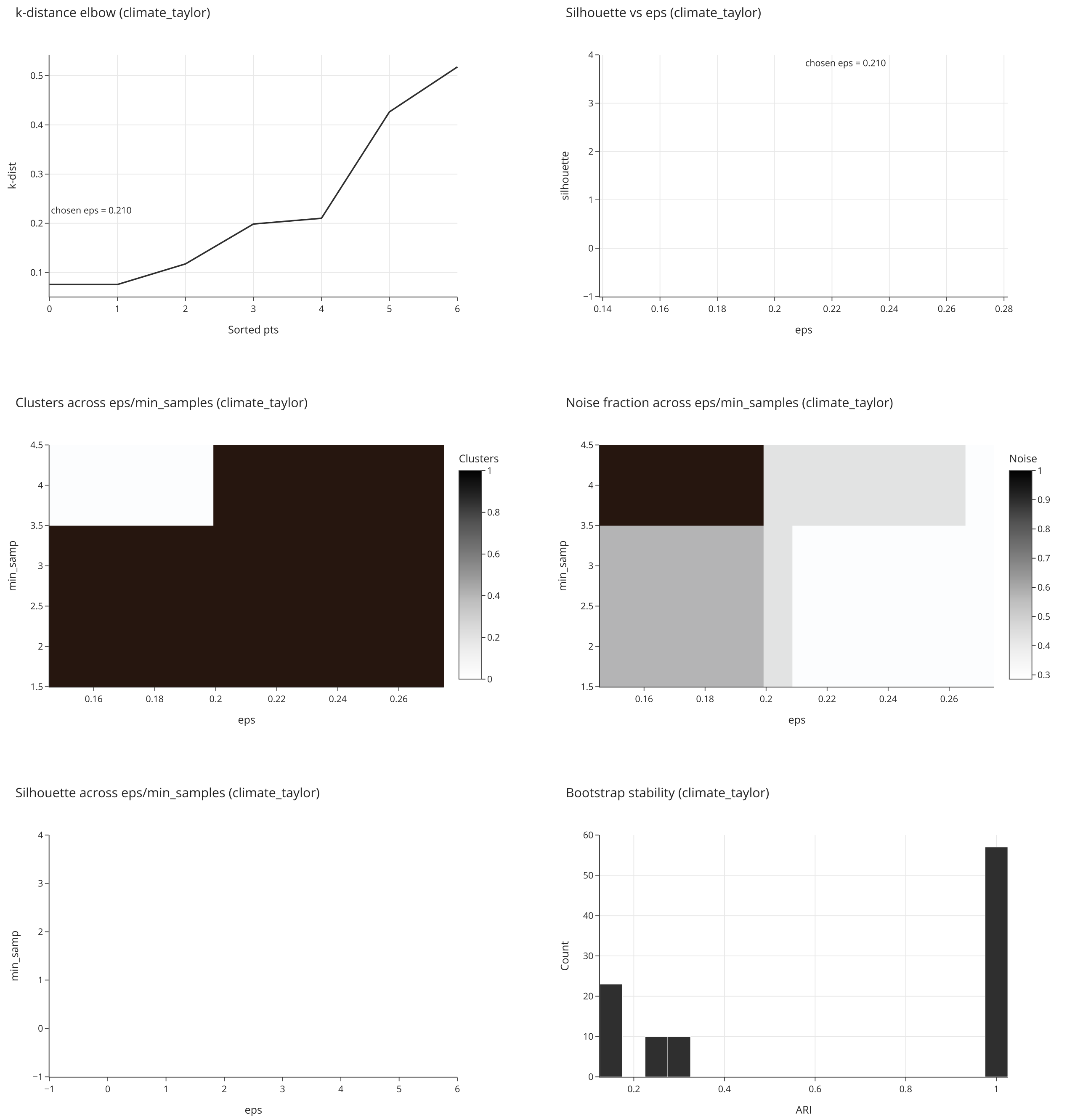

Figure S 1: **DBSCAN sensitivity analysis for the *Climate Model Comparison* example using Taylor diagram, related to section *Overview Panel Aggregation*.** The k-distance plot shows the automatically selected neighborhood radius ϵ. The sensitivity heatmaps summarize the number of clusters, the fraction of noise points, and the silhouette score across nearby DBSCAN parameter values. The bootstrap ARI histogram shows clustering stability under resampling.

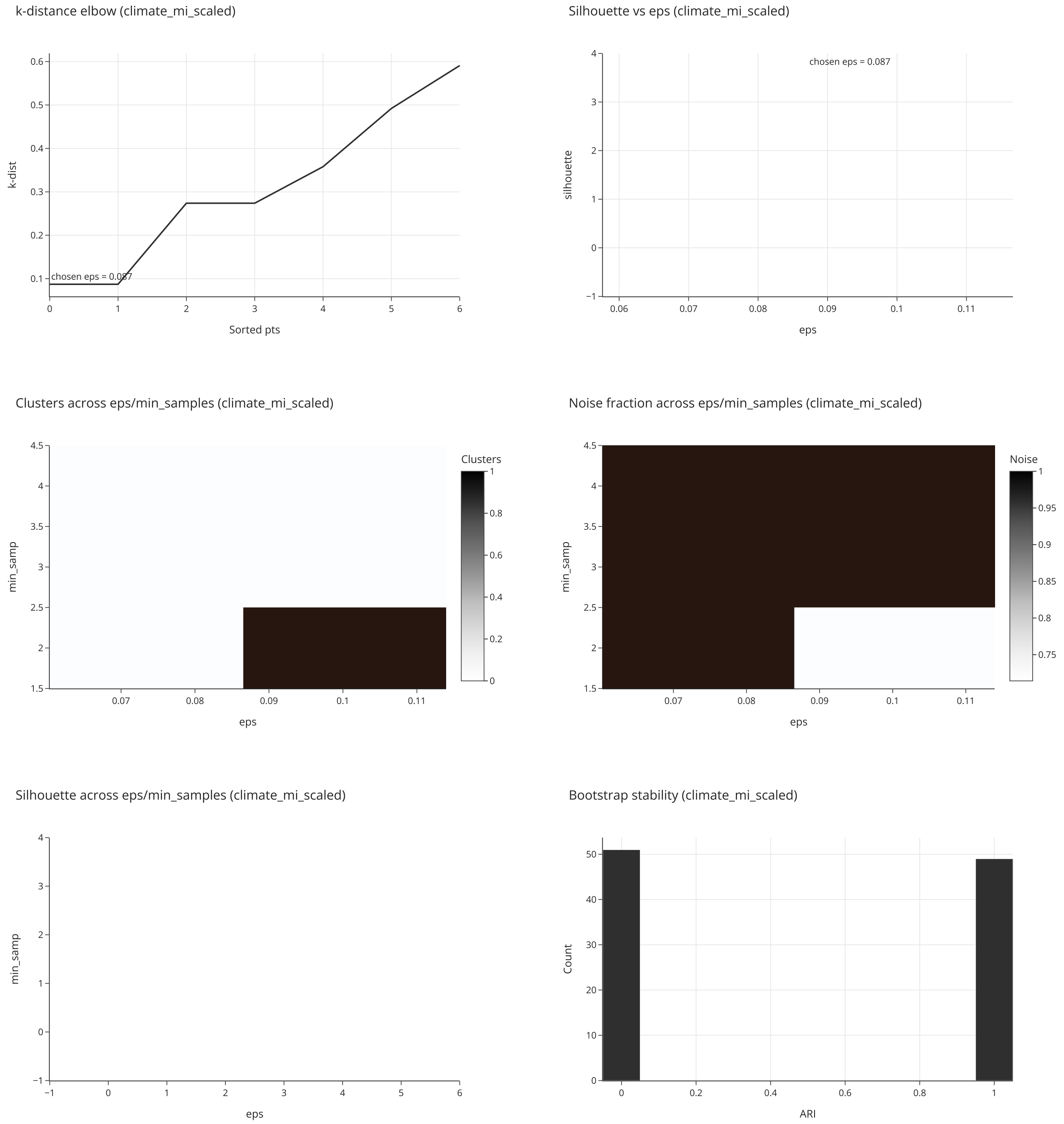

Figure S 2: **DBSCAN sensitivity analysis for the *Climate Model Comparison* example using Scaled Mutual Information diagram, related to section *Overview Panel Aggregation*.** The k-distance curve indicates the selected ϵ. The heatmaps show how cluster count, noise fraction, and silhouette score change across local parameter perturbations, and the bootstrap ARI distribution summarizes resampling stability.

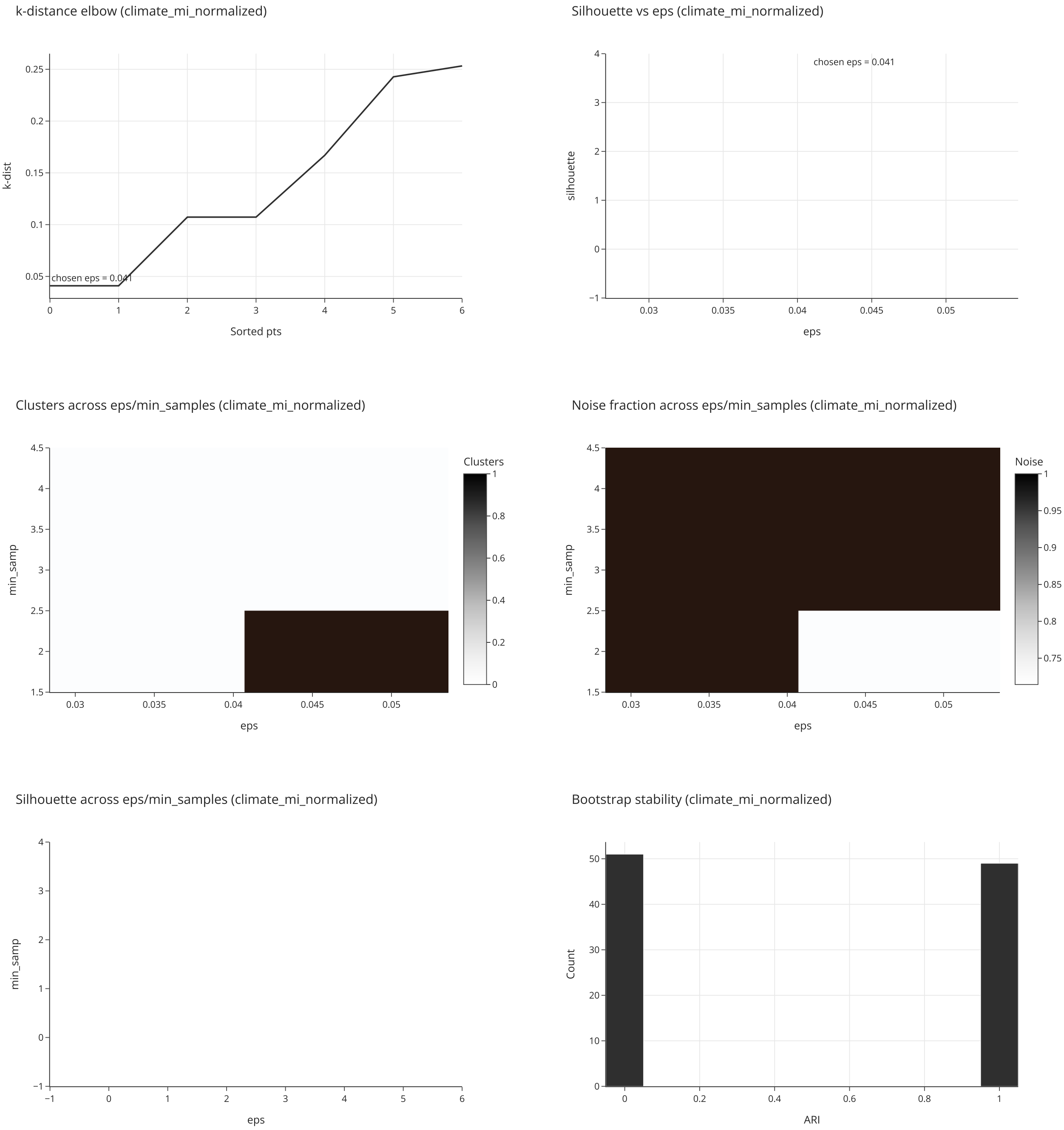

Figure S 3: **DBSCAN sensitivity analysis for the *Climate Model Comparison* example using Normalized Mutual Information diagram, related to section *Overview Panel Aggregation*.** The supplementary panels report the k-distance elbow used for automatic parameter selection, local sensitivity heatmaps for the number of clusters, noise fraction, and silhouette score, and the bootstrap ARI distribution for stability assessment.

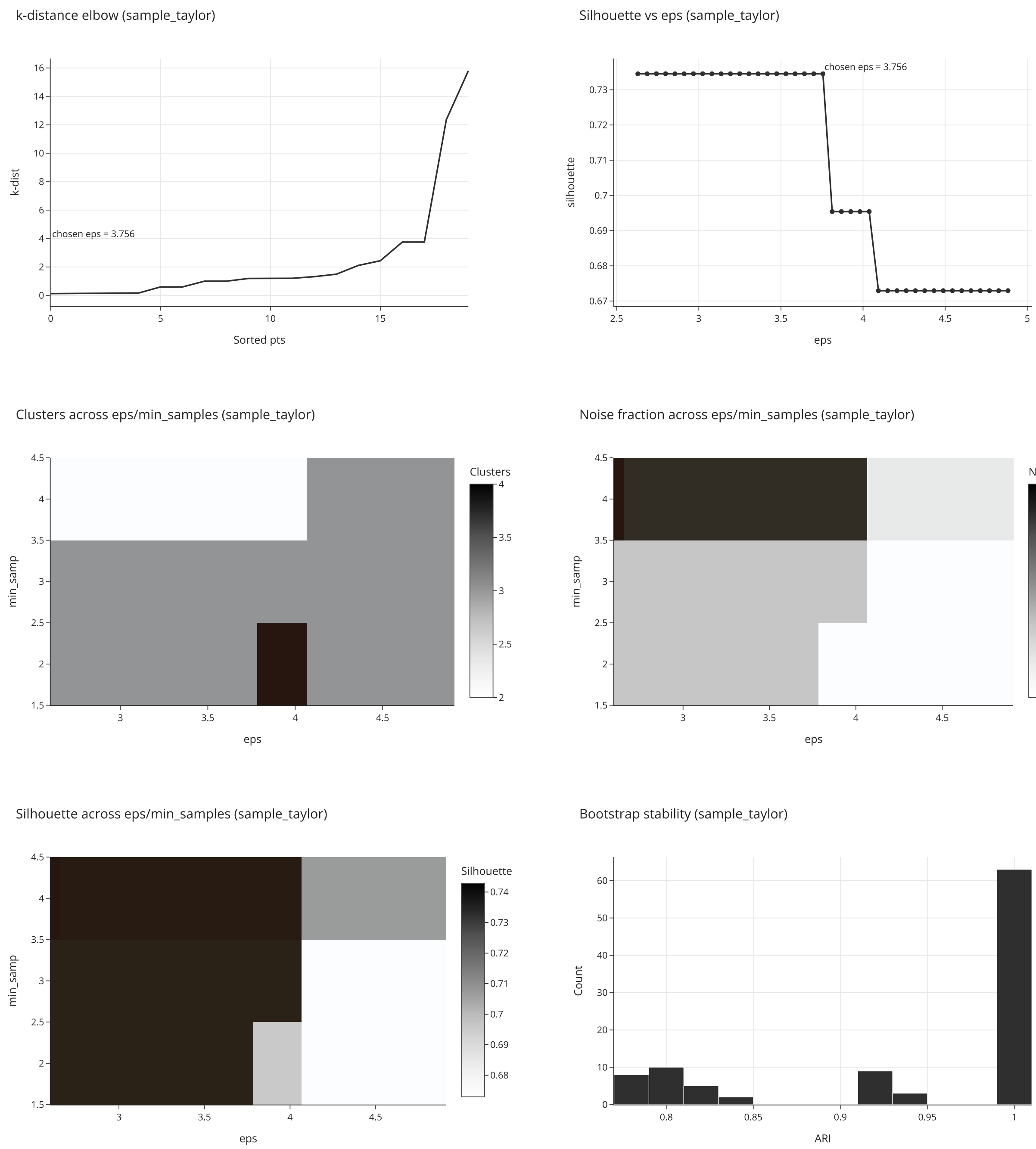

Figure S 4: **DBSCAN sensitivity analysis for the *Sample Analysis* example using Taylor diagram, related to section *Overview Panel Aggregation*.** This figure reports the selected k-distance elbow, the local behavior of cluster count, noise fraction, and silhouette score, and the resampling stability measured by bootstrap ARI.

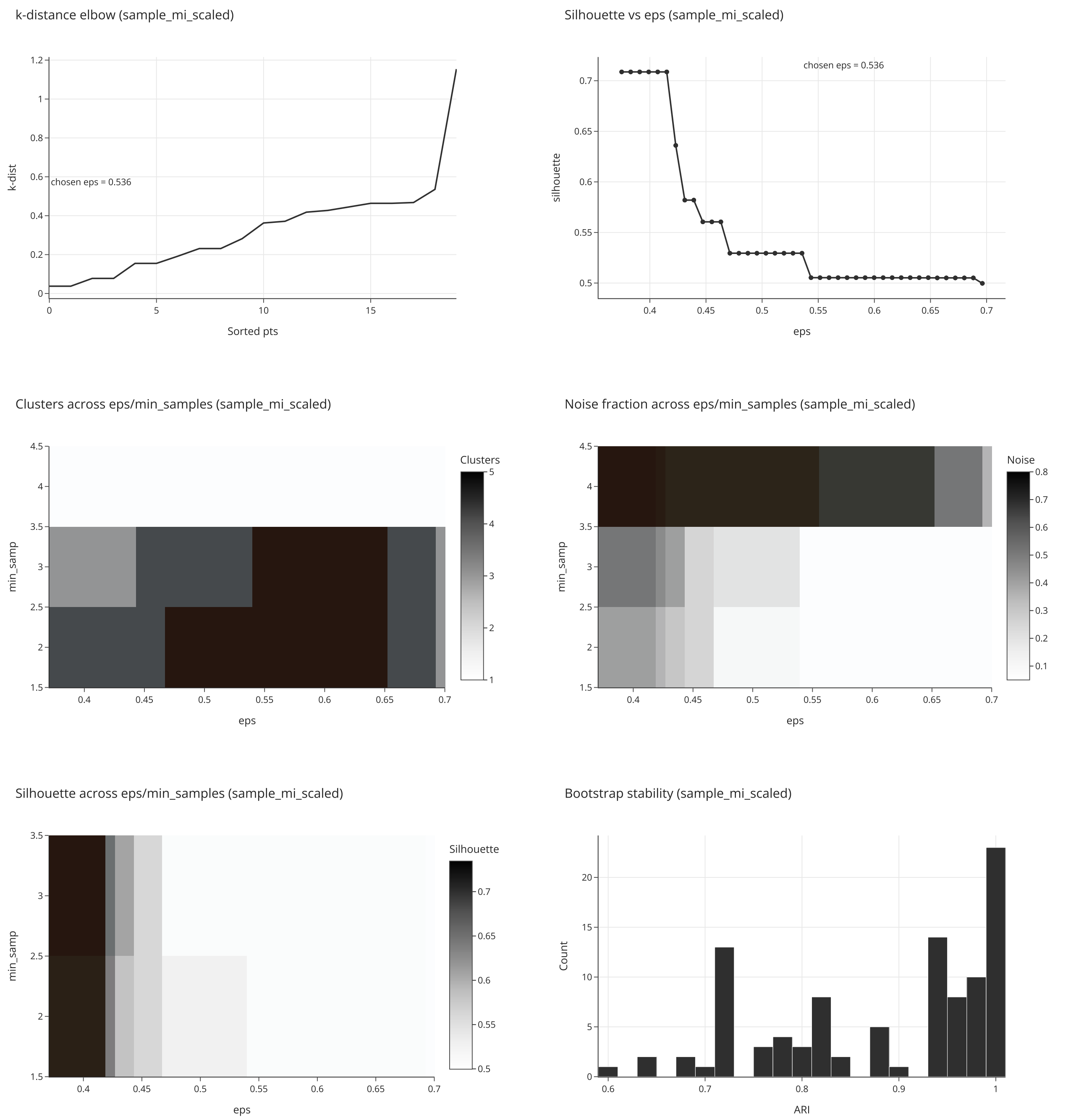

Figure S 5: **DBSCAN sensitivity analysis for the *Sample Analysis* example using Scaled Mutual Information diagram, related to section *Overview Panel Aggregation*.** The supplementary panels summarize the automatic parameter selection, parameter sensitivity, and bootstrap stability for the DBSCAN clustering.

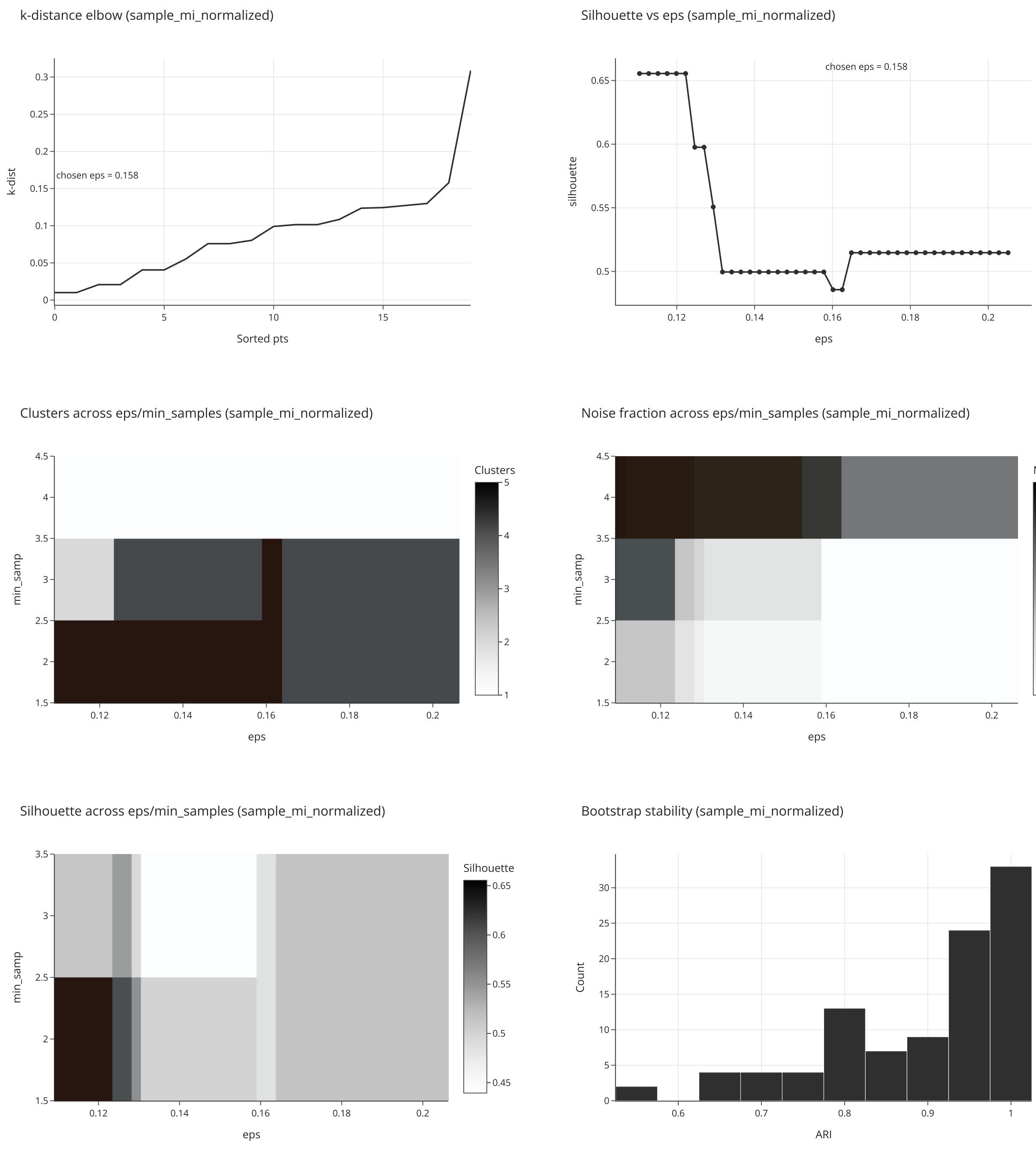

Figure S 6: **DBSCAN sensitivity analysis for the *Sample Analysis* example using Normalized Mutual Information diagram, related to section *Overview Panel Aggregation*.** The k-distance plot, sensitivity heatmaps, and bootstrap ARI distribution provide evidence that the selected DBSCAN parameters are stable in a local neighborhood of the chosen solution.

Table S 1: **Sensitivity of DBSCAN hyperparameters across datasets and reference models, related to Table 4.** For each data set–diagram type combination, the table reports the selected ϵ (eps0) and `min_samples0`, the resulting number of clusters, numbers of noise, core, and border points, the noise fraction, internal validation indices (silhouette, Davies–Bouldin, Calinski–Harabasz), and bootstrap ARI summary statistics. Missing values (`NaN`) for the internal indices occur in cases where DBSCAN produced an effectively single-cluster or otherwise degenerate solution, for which these cluster validity measures are undefined or not meaningful.

dataset_name	n_input_rows	eps0	min_samples0	n_clusters	n_noise	noise_frac	n_core	n_border
sample_taylor	21	3.755718	2	3	4	0.200000	16	0
sample_mi_scaled	21	0.535538	2	5	2	0.100000	18	0
sample_mi_normalized	21	0.157772	2	5	2	0.100000	18	0
climate_taylor	8	0.210160	2	1	2	0.285714	5	0
climate_mi_scaled	8	0.087172	2	1	5	0.714286	2	0
climate_mi_normalized	8	0.040984	2	1	5	0.714286	2	0
ml_taylor	12	0.390163	2	1	1	0.090909	10	0
ml_mi_scaled	12	0.459780	2	1	1	0.090909	10	0
ml_mi_normalized	12	0.200590	2	1	2	0.181818	9	0

dataset_name	silhouette	davies_bouldin	calinski_harabasz	bootstrap_ari_mean	bootstrap_ari_std	bootstrap_ari_median
sample_taylor	0.734550	0.328345	74.015206	0.941612	0.084816	1.000000
sample_mi_scaled	0.529502	0.514737	42.072164	0.881381	0.115594	0.937500
sample_mi_normalized	0.499542	0.536691	42.000493	0.892394	0.113946	0.937500
climate_taylor	NaN	NaN	NaN	0.659982	0.394792	1.000000
climate_mi_scaled	NaN	NaN	NaN	0.490000	0.502418	0.000000
climate_mi_normalized	NaN	NaN	NaN	0.490000	0.502418	0.000000
ml_taylor	NaN	NaN	NaN	0.594593	0.394017	0.461538
ml_mi_scaled	NaN	NaN	NaN	0.816525	0.321336	1.000000
ml_mi_normalized	NaN	NaN	NaN	0.879249	0.230005	1.000000

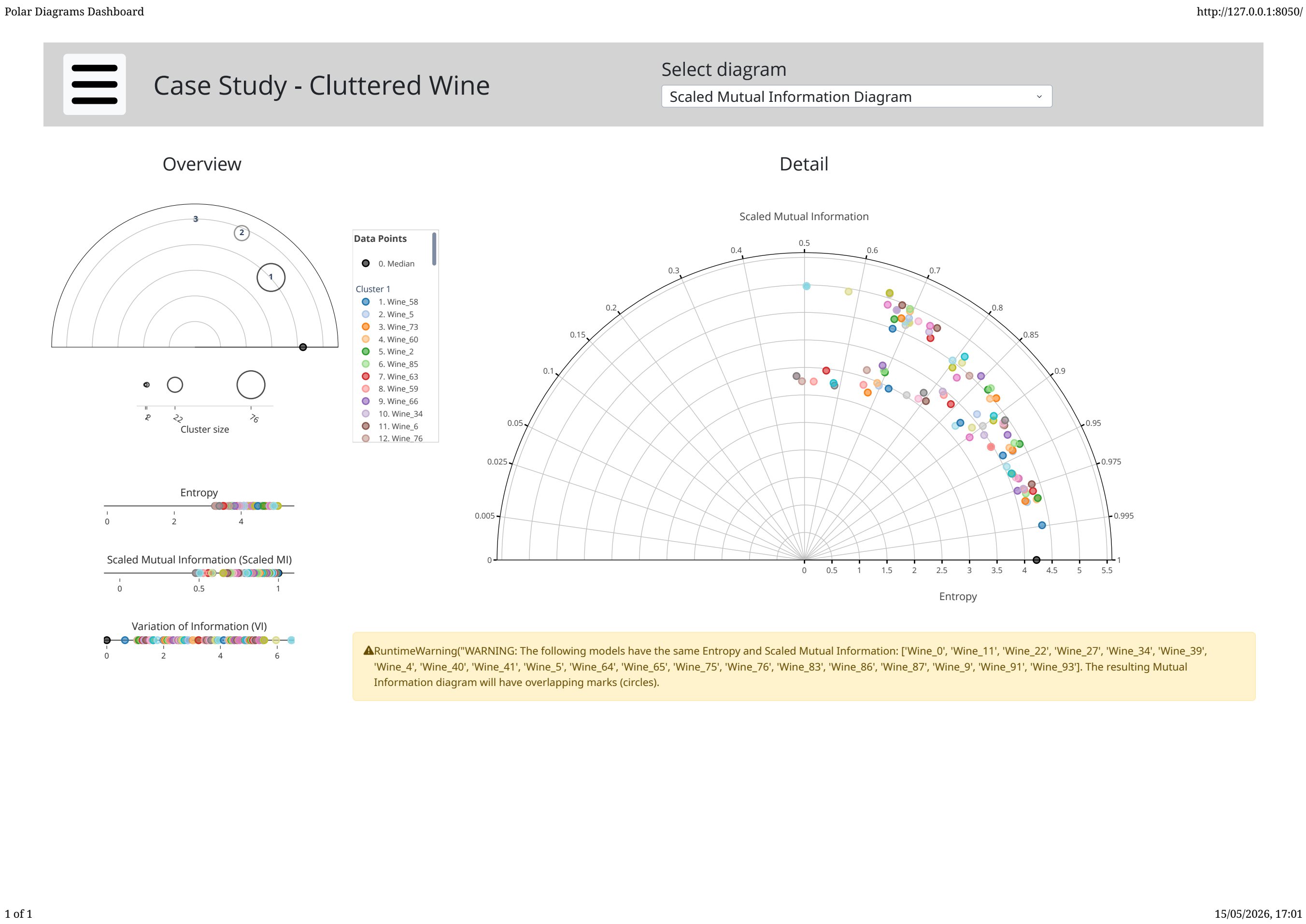

Figure S 7: **Screenshot of the proposed dashboard showing 100 models/points.** The figure illustrates the scalability limits of the approach, where visual clutter increases and color reuse becomes necessary due to the limited set of distinguishable colors. In this scenario, the legend is no longer sufficient for reliable identification, and users must rely on interactive elements such as tooltips to distinguish individual models.

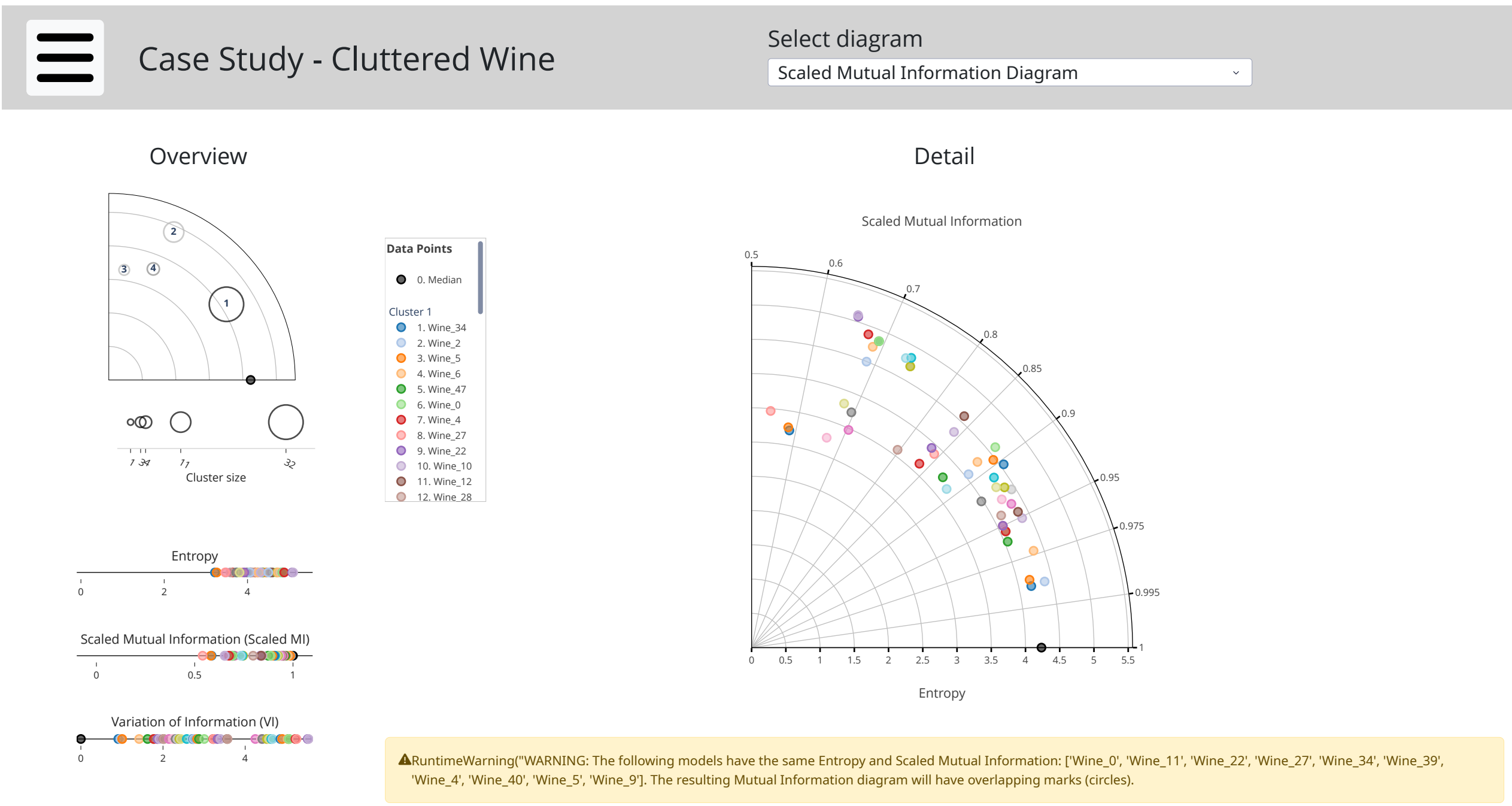

Figure S 8: **Screenshot of the proposed dashboard showing 50 models/points.** While the level of visual clutter is reduced compared to larger data sets, color reuse may still occur, and careful interaction is required to accurately identify individual models. This figure demonstrates a moderate scalability scenario.

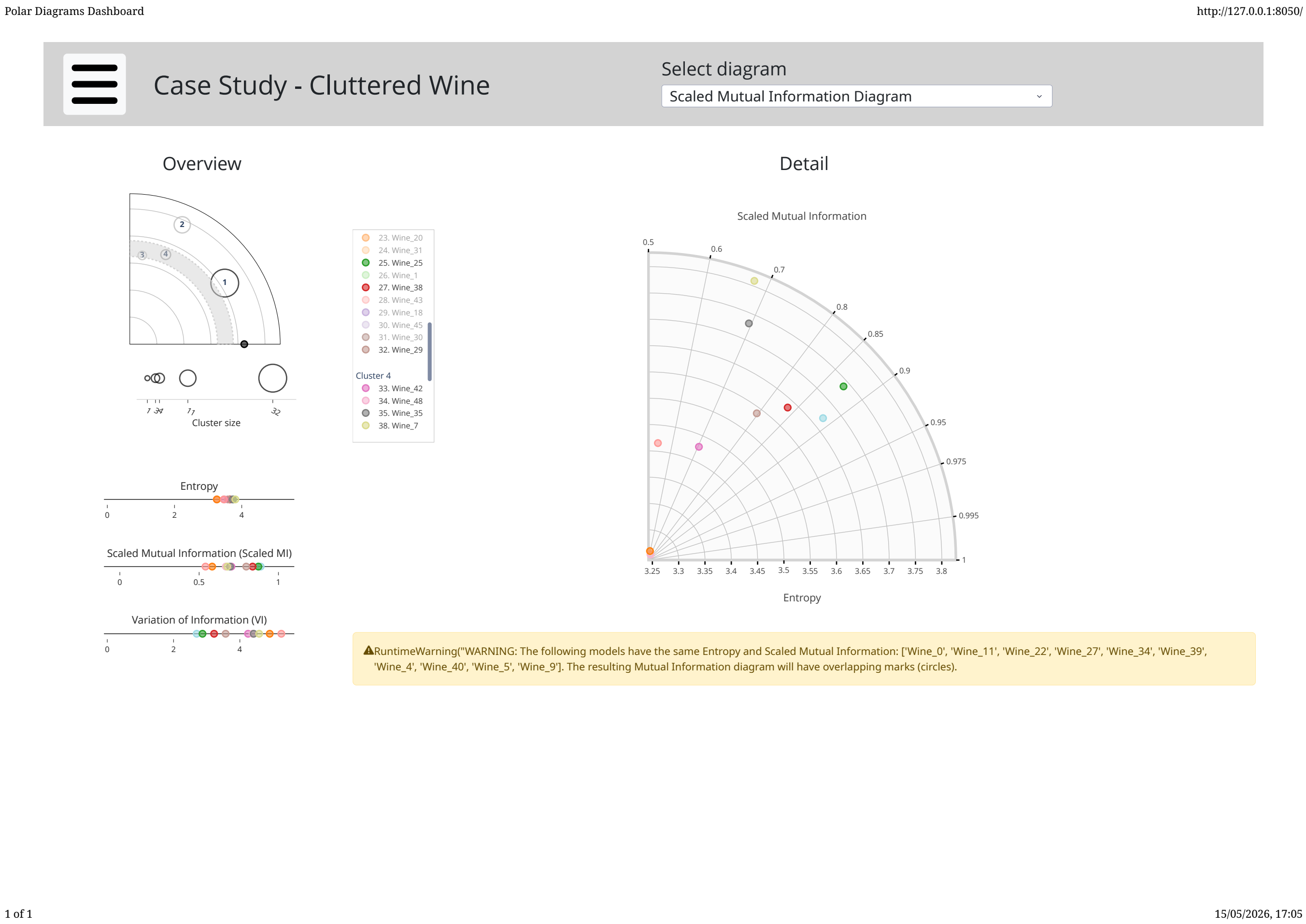

Figure S 9: **Screenshot of the proposed dashboard showing 50 models/points with a selected polar region of interest.** By focusing on a subset of the data, the approach reduces perceived visual clutter and supports more effective analysis. The overview+detail interaction enables users to inspect specific regions while maintaining contextual awareness.
